# Ribosome-associated proteins in fungal ribosome homeostasis: Conceptual opportunities for peptide-based modulation

**DOI:** 10.71150/jm.2511006

**Published:** 2026-02-24

**Authors:** Yongjun Kim, Chang-Jun Ji, Seohyun Park, Junsuk Lee, Jiwoon Jung, Yejin Kim, Dabin Pyeon, Yoon-Mo Yang

**Affiliations:** 1 Graduate School of Biomedical Science & Engineering, Hanyang University, Seoul 04763, Republic of Korea; 2 Department of Life Science and Research Institute for Natural Sciences, Hanyang University, Seoul 04763, Republic of Korea; 3 Hanyang Institute of Bioscience and Biotechnology, Hanyang University, Seoul 04763, Republic of Korea

**Keywords:** ribosome homeostasis, ribosomal protein, antifungal peptide

## Abstract

Ribosomes are essential macromolecular machines that facilitate protein synthesis and have long been recognized as effective targets for antimicrobial agents. While structural differences between prokaryotic and eukaryotic ribosomes form the basis for selective antibiotics against bacteria, similar approaches for developing antifungal agents targeting ribosomes have remained limited due to the high sequence and structural conservation with human ribosomes. However, emerging insights into ribosome homeostasis, including ribosome biogenesis, turnover, and hibernation, have uncovered a set of ribosome-associated proteins whose function is critical yet display greater sequence divergence from their human counterparts. These observations suggest that these regulatory components may represent viable antifungal targets by disrupting fungal proteostasis. The present review aims to explore this developing concept by examining ribosome-associated factors and considering whether short ribosomal protein-derived peptides may eventually serve as druggable molecules for selectively modulating these pathways in fungal pathogens.

## Introduction

Alongside pathogenic bacteria, fungal pathogens are emerging as a significant and growing public health threat worldwide (Casalini et al., 2024). Despite growing interest in the biological role of fungi in the human microbiome (Chung et al., 2024), pathogenic fungi such as *Candida*, *Cryptococcus*, and *Aspergillus* species are reported as major causes of invasive infections, particularly in immunocompromised patients (Brown et al., 2012). The most effective method for controlling pathogenic fungi remains the use of antifungal agents, and indeed, these drugs have played a pivotal role in diagnostic and therapeutic settings (Souza et al., 2025). Antifungal agents currently used clinically are classified into three major mechanisms based on their molecular targets: cell membrane-targeting drugs (e.g., azoles, polyenes), cell wall synthesis inhibitors (e.g., echinocandins), and nucleotide synthesis inhibitors (e.g., flucytosine) (Mazu et al., 2016). While these drug classes are clinically effective, the persistent rise in drug resistance poses a serious problem (Souza et al., 2025). Resistance primarily arises through core target alterations or the activation of compensatory stress adaptation programs (Cowen et al., 2014). For example, azole and polyene resistance is associated with ERG11 mutations, ergosterol pathway rerouting, or increased efflux pump activity (Flowers et al., 2015), while echinocandin resistance is linked to cell wall remodeling, including FKS hotspot mutations and increased chitin production (Perlin, 2015). In contrast, flucytosine resistance primarily stems from impairment of the FCY2–FCY1–FUR1 pathway, demonstrating that different selective pressures act depending on the target characteristics (Papon et al., 2007).

Protein translation is essential for the survival of all living organisms and is considered a potential antifungal target. However, to date, the only FDA-approved antifungal drug directly targeting protein synthesis is tavaborole, a fungal leucyl-tRNA synthetase inhibitor (Zhang and Ma, 2019). Sordarin, once considered a systemic therapeutic candidate, strongly inhibits eukaryotic elongation factor 2 (eEF2) (Justice et al., 1998) but development was halted due to in vivo efficacy and pharmacologic constraints (Odds, 2001).

In contrast, ribosomes have long been a successful target in the antimicrobial field. Several drugs are clinically used against bacteria, including kanamycin and tetracycline (30S targets), chloramphenicol and erythromycin (50S targets), and biomycin (both) (Hutchings et al., 2019; Yonath, 2005), and recently, lasso peptide-based antimicrobials synthesized within the ribosome that selectively inhibit it have also been reported (Jangra et al., 2025). However, approaches directly targeting fungal ribosomes remain challenging. The high structural and sequence conservation between fungal and human ribosomes makes it difficult to identify targets that simultaneously satisfy selectivity and toxicity criteria (Melnikov et al., 2012).

Interestingly, recent studies have begun to show that pathways maintaining ribosomal homeostasis such as ribosome biosynthesis, ribosome hibernation, and selective ribosome turnover (i.e., ribophagy) are important for cellular proteostasis (Prossliner et al., 2018; Tye et al., 2019; Wyant et al., 2018). Taken together, these findings suggest that maintaining ribosome equilibrium is essential for cell survival, strategies that prevent cells from properly regulating their ribosome numbers may represent alternative approach for suppressing microbial growth. In fungi, for example, the number of ribosomes correlates closely with growth rate and virulence (Bhabhra and Askew, 2005; Jorgensen et al., 2004). Importantly, proteins acting in these pathways (i.e., ribosome-associated proteins) are reported to be more heterogeneous compared to their human homologs. These observations suggest that ribosome-associated proteins mediating ribosome homeostasis may represent a novel target space distinct from existing antifungal mechanisms. This strategy could be an intriguing approach, particularly as it may circumvent long-term selective pressure arising from existing drug classes. Below, we outline three key pathways maintaining ribosomal homeostasis in fungi (ribosomal biosynthesis, ribosomal hibernation, and ribophagy), followed by a conceptual exploration of proteins functioning in these processes from the perspective of their targetability based on ribosomal protein (RP)-derived peptides.

## Pathways of Maintaining Ribosome Homeostasis in Fungi

**Fig. 1.**
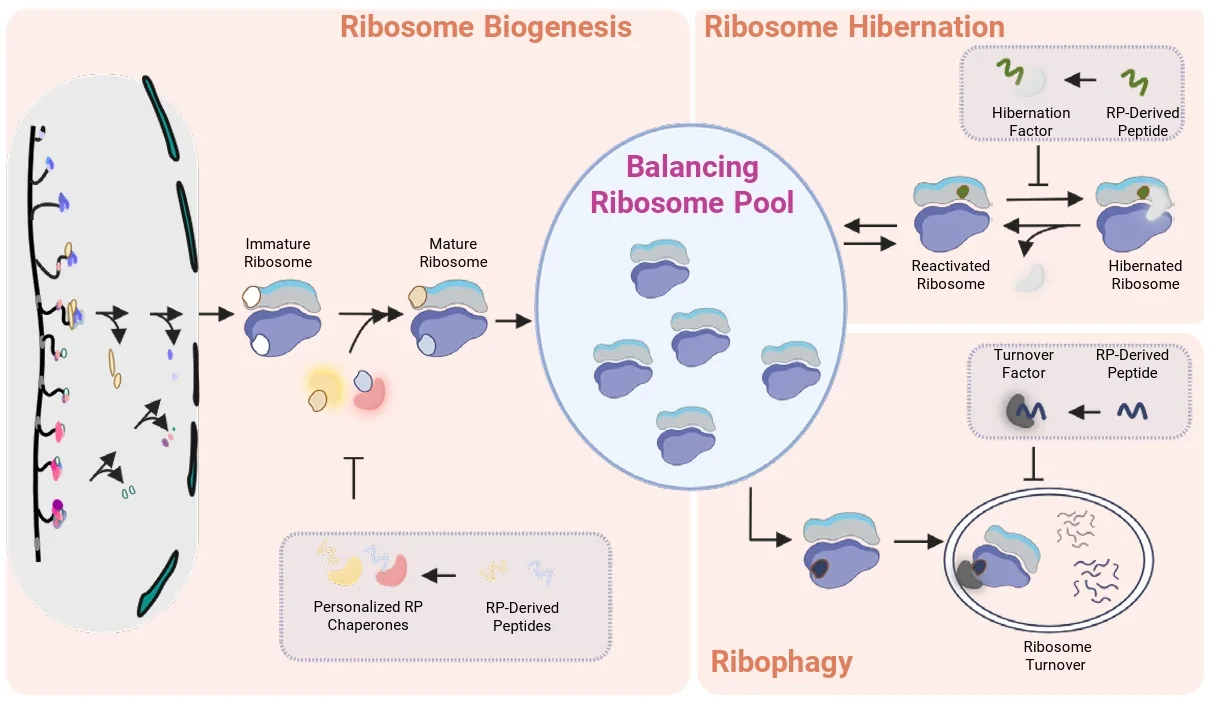
Schematic view illustrating how cells maintain ribosome homeostasis and strategies to disrupt by RP-derived peptides. Three key processes that balance the active ribosome pool are depicted. Ribosome biogenesis involves stepwise maturation through the incorporation of ribosomal proteins, many of which require personalized RP chaperones for proper delivery and assembly. Disruption of these chaperones impairs RP incorporation, ultimately leading to an insufficient number of functional ribosomes. Ribosome hibernation depends on specific hibernation factors that regulate ribosome activity under stress conditions; inactivation of these factors results in inefficient control of ribosome pool balance. Ribophagy selectively degrades ribosomes in response to specific stress, and disruption of ribosome turnover factors leads to dysregulated stress responses and impaired ribosome homeostasis.

Studies have revealed that newly assembled ribosomes undergo multiple quality control steps to ensure their structural integrity and functionality before entering the translation pool (Fig. 1) (Parker and Karbstein, 2023). Moreover, partially functional ribosomes can incur collisions, leading to their decay (Parker et al., 2024). In addition, oxidatively damaged mature ribosomes are recognized and repaired (Yang and Karbstein, 2024a). These mechanisms are thought to protect cells by avoiding defective ribosomes in the translating “ribosome pool”. Conversely, evidence suggests that the total number of ribosomes is equally important, as protein synthesis depends on the available ribosome concentrations in cells (Ivanov et al., 2022; Mills and Green, 2017). Together, these findings suggest that disrupting ribosome homeostasis can compromise fungal adaptation to stress and impair survival (Tye et al., 2019; Warner, 1999). Therefore, we will first discuss three important pathways related to ribosome homeostasis, including ribosome biogenesis, ribophagy, and ribosome hibernation, which can be leveraged to modulate intracellular translation without directly targeting ribosomes.

### Overview of ribosome biogenesis and the role of personalized RP chaperones

In eukaryotes, ribosomes are composed of two subunits referred to the small 40S and the large 60S subunit, which together contains four ribosomal RNAs (rRNAs) and approximately 79 RPs. To generate this large macromolecular complex ribosome assembly starts in the nucleolus with the transcription of rRNA precursors, followed by simultaneous folding, processing and modification of the rRNA (Henras et al., 2015). These maturation events of rRNA are coupled to the hierarchical binding of RPs (de la Cruz et al., 2015). To manage this complicated process precisely, ribosome biogenesis is orchestrated by a large set of assembly factors, which facilitate rRNA maturation, proper RP incorporation, structural remodeling, and rigorous quality control before joining the translating “ribosome pool” (Fig. 1) (Klinge and Woolford, 2019; Parker and Karbstein, 2023).

The process of ribosome biogenesis is largely conserved from yeast to human (Ameismeier et al., 2018; Fiorentino et al., 2025; Tomecki et al., 2017; Wild et al., 2010), together with the assembly factors as well (Dorner et al., 2023). Out of ~200 assembly factors identified, a subset of chaperone proteins has been classified based on their function. While RPs are relatively small and basic proteins, they are prone to aggregate when they fail to incorporate into ribosomes (Gorenstein and Warner, 1977; Sung et al., 2016; Warner, 1977). To prevent misfolding and ensure efficient ribosome assembly of these RPs, subset of “personalized” chaperones have been identified in yeast, interacting with one or more of 13 specific RPs (Table 1) (Iouk et al., 2001; Koch et al., 2012; Kressler et al., 2012; Mitterer et al., 2016; Pillet et al., 2015; Schutz et al., 2014; Stelter et al., 2015; Ting et al., 2017; West et al., 2005; Yang and Karbstein, 2022). While most of these personalized RP chaperones have human homologs (Yang et al., 2023), due to its substantial study performed in yeast, it remains unclear whether all these human homologs retain the same client specificity as their yeast counterparts.

Because these personalized chaperones bind and stabilize their RP clients and deliver them to newly synthesizing ribosomes (Pillet et al., 2017), they contribute to maintaining proper ribosome stoichiometry (Kressler et al., 2012). In *S. cerevisiae*, genetic deletion studies revealed that among the 11 personalized chaperones, four are essential (inviable null phenotype), while six cause significant growth defects (Table 1), many of which can be rescued by overexpression of their client RPs (Table 1). Together, these findings suggest that impairing the function of these personalized RP chaperones can limit the number of functionally mature ribosomes that enter the “ribosome pool”.

### Overview of ribophagy and the role of autophagy-related proteins

Ribophagy is an autophagy pathway in which ribosomes are selectively degraded (Kraft et al., 2008). In mammalian cells, ribophagy is facilitated by a specific cargo receptor NUFIP1. While NUFIP1 localizes to the nucleus under nutrient-rich conditions, during starvation it translocates to the cytoplasm where it directly binds to ribosomes and delivers them to autophagosomes. This is facilitated by an interaction with LC3B/ATG8, a core autophagosome component for selective degradation of ribosomes. Lack of NUFIP1 leads to impaired ribophagy flux and reduces the availability of nucleotides driven from rRNA degradation, which ultimately compromises cell survival under nutrient-limiting conditions (Wyant et al., 2018). Despite these findings, other studies have questioned the universal role of NUFIP1 during ribophagy. For instance, in cancer-associated fibroblasts NUFIP1 participates primarily in rRNA degradation and not degradation of RPs, suggesting context-dependent roles for NUFIP1 in ribosome turnover (Yuan et al., 2022). Moreover, yeast lack an identifiable homolog of NUFIP1 implying an additional evolutionarily conserved mechanism might govern ribophagy across eukaryotic species. Indeed, recent findings have identified Rpl12 as a potential ribophagy receptor conserved across eukaryotes (Chen et al., 2025; Tutak and Karbstein, 2025). It was shown that Rpl12 directly interacts with key autophagy-related proteins Atg8 and Atg11 through its N-terminal and central domains, respectively.

Disrupting essential components of the ribophagy machinery have significant consequences for ribosome homeostasis (Chen et al., 2025). Disruption of ribophagy may preserve ribosome number; however, accumulation of defective ribosomes could ultimately reduce translational fidelity and increase cellular stress (Parker and Karbstein, 2023; Yang and Karbstein, 2024a), particularly under nutrient limitation or host-like stress conditions where ribophagy becomes essential for survival (Chen et al., 2025). Moreover, impairing ribophagy limits the ability to recycle rRNA-derived nucleotides and amino acids, a process that supports survival during starvation (An and Harper, 2020; Wyant et al., 2018). Together, these effects suggest that inhibiting Atg8 or Atg11 dependent ribophagy may lead to an accumulation of unnecessary “ribosome pool”, potentially reducing fungal fitness in environments requiring rapid adaptation.

### Overview of ribosome hibernation and the role of hibernation factors

Ribosome hibernation is an evolutionarily conserved mechanism, in which cells actively transition ribosomes into a translationally inactive state in response to environmental stresses including nutrient starvation, oxidative damage, or energy limitation (Prossliner et al., 2018; Van Dyke et al., 2006; Wang et al., 2018; Wells et al., 2020).

In yeast, key regulators for ribosome hibernation have been identified. The first factor is Stm1, which binds to 80S ribosomes under nutrient-depleted conditions and stabilizes ribosomes in an inactive conformation, preventing their dissociation and degradation. Upon nutrient repletion, Stm1 is phosphorylated by TORC1, triggering its dissociation and allowing translation to resume (Shetty et al., 2023). In addition to Stm1, Lso2 was also identified as a hibernation factor which binds both the A and P sites of 80S ribosomes, sterically blocking translation initiation and elongation (Wang et al., 2018; Wells et al., 2020). Functional homologs of these proteins have been identified in mammalian cells as well (Wells et al., 2020). The Stm1 homolog SERBP1 and the Lso2 homolog CCDC124 ensure ribosome preservation and contribute to the formation of a hibernating “ribosome pool” that can be reactivated. Transition of ribosomes into this dormant configuration advantages cells to reduce energy expenditure and protect ribosomes from degradation or damage. In addition, this process is reversible which allows cells to rapidly resume translation once more favorable conditions arise. Therefore, in pathogenic fungi, disrupting these hibernation factors could destabilize the dormant “ribosome pool” and impair recovery from host-induced stresses, thereby attenuating growth and virulence.

## Ribosome Homeostasis as an Antifungal Target Space

The section above outlines the rationale for targeting pathways that regulate ribosome homeostasis as an alternative to directly inhibiting the ribosome itself to affect fungal physiology. That is, disrupting these pathways can eventually destabilize protein homeostasis and impair the ability of fungi to adapt to fluctuating environments. Notably, although these pathways are conserved across eukaryotes, key factors required to this process either lacks mammalian homologs or share relatively low sequence conservation, revealing the space to explore them as novel antifungal targets (Tables 1 and 2). In the following sections, we will discuss how antimicrobial peptides (AMPs) inspired by RP-derived peptides may serve as a promising starting point to selectively target these ribosome-associated proteins.

### RP-derived peptides as a novel antifungal AMP modality

While utilizing RP-derived peptides as antifungal agents conceptually align with strategies based on AMPs (Kang et al., 2017; Lee et al., 2023; Ra and Bang, 2024; Ryu et al., 2021), yet they differ in both rationale and mode of action. Whereas many antifungal AMPs, including human defensins, primarily act through membrane disruption (Buda De Cesare et al., 2020), RP-derived peptides suggested here are designed to competitively interfere based on protein-protein interaction interfaces within ribosome-associated proteins important for ribosome homeostasis pathways. These mechanistic distinction positions of the peptides suggested here are not simply as another AMP subclass but as a targeted modality intended to exploit proteostasis vulnerabilities in translational regulation. Therefore, framing the approach within the broader AMP landscape highlights both its novelty and its potential to complement existing AMP classes rather than replicate their mechanisms.

### Targeting personalized RP chaperones

Interestingly, while RPs generally exhibit strong evolutionary conservation (> 60% sequence identity between human and yeast), the personalized RP chaperones themselves are more divergent, sharing only ~30% sequence identity. This divergence suggests that targeting these chaperones may provide an antifungal strategy with reduced risk of off-target effects in humans. Moreover, several personalized RP chaperones, including Yar1, Acl4, and Loc1, lack human homologs, making them amenable to peptide-based inhibitor design, especially given that the binding sites for Rps3 and Rpl4 are already characterized (Table 1).

**Fig. 2.**
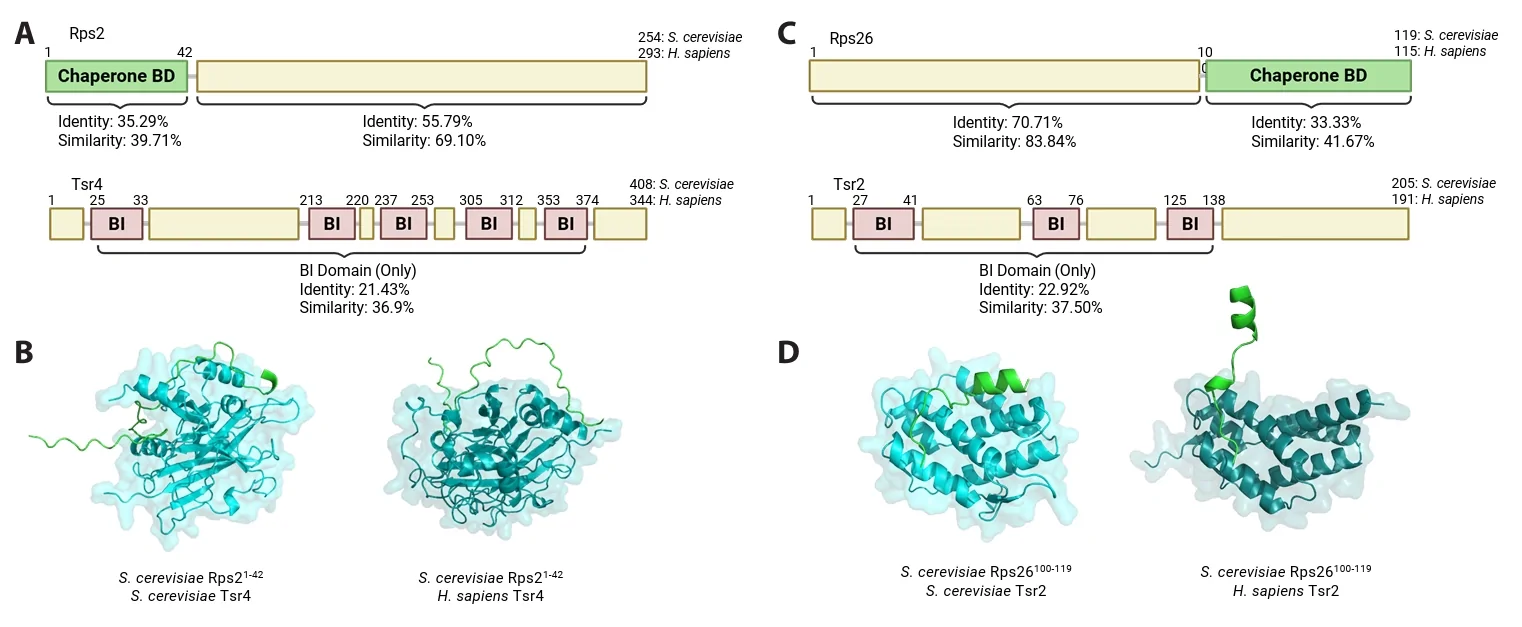
Targeting personalized RP chaperones by utilizing RP-derived peptides. (A) Sequence similarity and identity of Rps2 and Tsr4 between *S. cerevisiae* and *H. sapiens*. The chaperone-binding domain (BD) of the RP and the binding interface (BI) of the chaperone are indicated. (B) The structure of the *S. cerevisiae* Rps2 N-terminal region (green, residues 1–42) in complex with *S. cerevisiae* Tsr4 (left) or *H. sapiens* Tsr4 (right) was predicted by AlphaFold (Jumper et al., 2021). (C) Sequence similarity and identity of Rps26 and Tsr2 between *S. cerevisiae* and *H. sapiens*. The BD of the RP and the BI of the chaperone are indicated. (D) The structure of the *S. cerevisiae* Rps26 C-terminal region (green, residues 100–119) in complex with *S. cerevisiae* Tsr2 (left) was adapted from PDB 6G04 (Schutz et al., 2018). The structure of the *S. cerevisiae* Rps26 C-terminal region in complex with *H. sapiens* Tsr2 (right) was predicted by AlphaFold (Jumper et al., 2021).

A conceptual basis for targeting personalized RP chaperones also comes from the dominant-negative effect observed by an Rps2-derived peptide (Black et al., 2019). In a study originally aimed at mapping the Tsr4-binding domain on Rps2, overexpression of the N-terminal Rps2 fragment (sufficient for Tsr4 binding) in wild-type yeast caused growth defects, likely due to sequestration of the personalized chaperone Tsr4. Another study showed that this chaperone binding domain of Rps2 requires at least 42 amino acids at the N-terminus (Rossler et al., 2019), and this region of yeast Rps2 that mediates this interaction differs significantly from its human counterpart, suggesting that such peptides could act selectively on pathogenic fungi with less off-target binding to the host Tsr4 homolog (Fig. 2A–2B).

Similarly, multiple studies suggest that this strategy may also apply to defect fungal Tsr2 (Schutz et al., 2014, 2018; Yang and Karbstein, 2022, 2024b). Structural analysis and biochemical studies indicate that Tsr2 interacts with two distinct regions on Rps26, with one region including the C-terminal tail domain (Schutz et al., 2018). Interestingly, the sequence of this tail region varies between species, which may allow species specific interactions between Tsr2 and Rps26 (Fig. 2C–2D). Thus, if this C-terminal peptide of fungal Rps26 could competitively inhibit fungal Tsr2 by impairing its primary role in delivering Rps26 to maturate ribosomes, and consequently leading to the growth inhibition observed in Tsr2 null mutants (Table 1), it is expected to have a fungal-specific effect. Importantly, impairing Tsr2 function would not only disrupt ribosome biogenesis but could also compromise ribosome repair pathways, thereby increasing fungal sensitivity to oxidative stress (Yang and Karbstein, 2024a; Yang et al., 2023). Such vulnerabilities may, in turn, enhance the effectiveness of innate immune responses, which rely in part on oxidative stress.

### Targeting ribosome turnover factors

The selective autophagic degradation of ribosomes plays a critical role in maintaining cellular homeostasis, particularly under nutrient-limiting conditions. Chen and colleagues have recently identified Rpl12 as a universal ribophagy receptor mediating this process (Chen et al., 2025). Notably, they have shown Rpl12 directly interacts with two key autophagy components, Atg8 and Atg11. Given that deletion of Atg8 or Atg11 impairs growth (Table 1), Rpl12-derived peptides that interact with these proteins may potentially serve as antifungal agents. However, targeting Atg8 using Rpl12-based peptides may present challenges due to the evolutionary conservation of the interaction surface on Rpl12 (Table 1). The N-terminal region of Rpl12 that interacts with Atg8 contains two essential residues, Pro3 and Glu21, which are highly conserved across fungi and humans. Moreover, human LC3B (Atg8 homolog) shares strong sequence identity with yeast Atg8, suggesting that peptide targeting this interface could have unintended off-target effects in human cells.

Conversely, Atg11 may represent a promising antifungal target over Atg8. It has been demonstrated through a combination of structural and biochemical analyses that the central region of Rpl12 directly interacts with Atg11. Furthermore, the phosphorylation of Ser79 and Ser101 within Rpl12 significantly enhances this interaction. These findings indicate that a peptide designed from the central region of Rpl12, incorporating phospho-mimetic substitutions at Ser79 and Ser101 (e.g., aspartate or glutamate residues), has a potential to function as a high-affinity inhibitor of Atg11. And while this central region of Rpl12 is highly conserved between yeast and humans, mammals lack an identifiable Atg11 homolog and only contain a functional homolog FIP200 (Hara et al., 2008). Therefore, the Atg11-Rpl12 interaction presents a novel candidate for fungal-specific therapeutic targeting.

### Targeting ribosome hibernation

Cells lacking Lso2 exhibit translational defects during recovery from stationary phase, underscoring its essential role in regulating the translation machinery (Wang et al., 2018). Lso2 inactivates 80S ribosomes by occupying the central region adjacent to the tRNA binding sites and making contacts with highly conserved regions of rRNA including helices H43 and H44 (Wang et al., 2018; Wells et al., 2020). Yet, due to its binding site on ribosomes, Lso2 may not be amenable to targeting via RP-derived peptides.

**Fig. 3.**
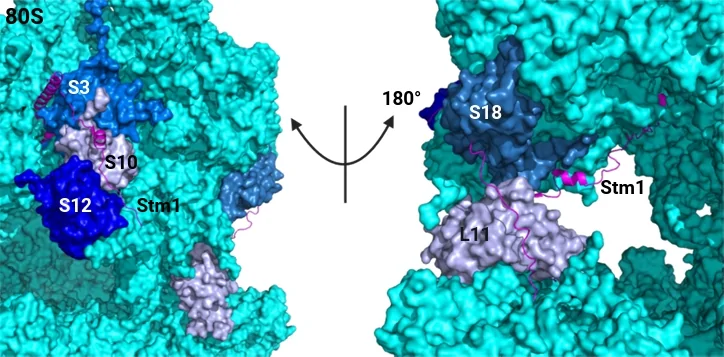
Structural location of RPs which interact with Stm1. Structural location of RPs that interact with Stm1. RPs (surface, blue) that make direct interactions with Stm1 (cartoon, magenta) are highlighted in multiple colors in space-filling models of the yeast 80S ribosome (surface, cyan, PDB ID 4V88) (Ben-Shem et al., 2011).

In contrast, Stm1 represents a more promising target for antifungal intervention. Stm1 functions by occupying the mRNA channel and stabilizing 80S complexes (Ben-Shem et al., 2011). In mammalian cells, Stm1 homolog SERBP1 performs a similar role, even though the overall sequence identity is relatively low (Tables 1 and 2). Stm1 is a 273-amino acid protein that interacts with a subset of both small and large subunit RPs. Structural analyses suggest that Stm1 makes direct contact with Rps3, Rps10, Rps12, Rps18, and Rpl11 (Fig. 3). Among these RPs, three specific domains seem critical for the association with Stm1 including Rps3^116–130^, Rpl11^28–48^, and Rps18^121–146^. These regions may serve as rational templates for the design of short competitive peptides that sequester Stm1 to disrupt its interaction with the ribosome. Since SERBP1 is the sole functional homolog of Stm1 in humans, and the Stm1/SERBP1 interaction with ribosomes is distinct, the use of RP-derived, Stm1-targeting peptides could effectively disrupt fungal ribosome hibernation while minimizing off-target effects on human ribosomes.

## Challenges and Limitations of RP-Derived Peptides as Antifungal Therapies

The strategy described above of using RP-derived peptides to selectively disrupt fungal ribosome homeostasis offers a conceptually attractive avenue for novel antifungal development. Yet, translating this concept into a viable therapeutic agent faces several other limitations inherent to peptide-based modalities, particularly in the context of fungal pathogens. Following discussion of these challenges is essential to ground the proposed approaches in a realistic translational context.

### Conceptual and mechanistic constraints

A conceptually similar strategy has been demonstrated using short peptides derived from 4EBP, which bind to eIF4E and lead to inhibition of ovarian cancer growth (Ko et al., 2009). This finding supports the broader feasibility of using structured peptide segments to selectively disrupt translation. However, direct demonstration of peptide base inhibition of ribosome-associated proteins to disrupt ribosome homeostasis has not yet been established. Thus, the strategy proposed here should currently be considered a conceptual approach grounded in structural biology. Empirical testing will be required to determine whether such peptides can achieve sufficient affinity, selectivity, and intracellular accessibility in fungal pathogens. Furthermore, these pathways are mediated by multi-component and spatially regulated complexes rather than a single obligate binary interaction, raising questions about whether RP-derived peptides would be sufficiently specific or competitive to perturb the targets ribosome binding in vivo given their intrinsic size limitation. In addition, the complexity and buffering capacity of the translation may allow fungi to compensate for partial inhibition, potentially necessitating multi-node targeting to achieve meaningful antifungal impact.

### Delivery and cellular penetration barriers

Despite the limitation of these peptides function efficiently to disrupt ribosome homeostasis, there are additional limitations that are required for discussion in terms of applying to AMP drugs (Xiao et al., 2025). First, limitation in cell wall and membrane permeability should be evaluated. A major challenge is the efficient intracellular delivery of peptides. Fungal cells possess a rigid cell wall that acts as a formidable barrier, followed by the cell membrane. While some AMPs have evolved mechanisms to breach these structures, the RP-derived inhibitory peptides proposed here are designed for intracellular PPI targeting and must therefore overcome both layers to reach their cytoplasmic targets (e.g., personalized chaperones or Stm1 or Atg11). Simple passive diffusion is generally ineffective for molecules like peptides, necessitating delivery strategies. To overcome these issues generating the most common way would be to generate a fusion peptide with cell-penetrating peptides. One positive view is the observation of CPP penetrating efficiency measured in *Candida* species (Gong and Karlsson, 2017). It was shown that the charge of CPP correlates with the penetrating efficiency, suggesting RP-derived peptides with strong positive charge may penetrate by itself, or at least support the penetrating efficiency. Yet combining with the CPP could also cause solubility issues, intracellular missing localization problems as well as decreased target specificity. Nanocarrier system strategies involving the conjugation or encapsulation of peptides within nanoparticles (NPs) would be an alternative method to apply for overcome peptide stability, improved bioavailability, and enabling targeted delivery to the infection site.

### Proteolytic instability and short half-life

Proteolytic instability and short half-life are also crucial for developing AMPs (Xiao et al., 2025). Peptides are inherently susceptible to degradation by ubiquitous proteases found in biological fluids, host tissues, and within the fungal cell itself. This proteolytic instability leads to a severely restricted systemic half-life and reduced bioavailability at the site of infection. To achieve therapeutic efficacy, peptide stability must be significantly enhanced, often through chemical modifications such as introducing D-amino acids or cyclization methods could be considered.

## Conclusion

This review highlights the roles of ribosome-associated proteins involved in ribosome biogenesis, ribophagy, and hibernation and illustrates how short RP-derived peptides can act as dominant-negative inhibitors of these processes. Such an approach holds the potential to impair ribosome homeostasis in pathogenic fungal and reduce virulence. However, significant limitations remain to be overcome before RP-derived peptides can be utilized as future antifungal drugs. This includes the delivery and stability of such peptides and enhancing their pharmacokinetics and bioavailability. Future studies integrating structural biology, genetics, and peptide engineering will be necessary for the success of this antifungal drug development.

## Figures and Tables

**Table 1.** Ribosomal proteins and ribosome-associated proteins in *S. cerevisiae* and *H. sapiens*

	Target (*S. cerevisiae*)	Null	Target homolog (Human)	Target similarity (Identity)	RP partner	RP partner similarity (Identity)	Recognition site	Reference
**Ribosome biogenesis**	Yar1 (22.36 kDa)	Growth defect	-		Rps3 (26.50 kDa)	78.8% (66.8%)	N-terminal	Koch et al. (2012); Mitterer et al. (2016)
	Tsr4 (45.99 kDa)	Inviable	PDCD2 (38.59 kDa)	40.2% (26.2%)	Rps2 (27.45 kDa)	62.5% (51.2%)	N-terminal	Black et al. (2019); Rossler et al. (2019)
	Tsr2 (23.71 kDa)	Growth defect	TSR2 (20.89 kDa)	44.4% (29.3%)	Rps26 (13.51 kDa)	76.4% (64.2%)	C-terminal+middle domain	Schutz et al. (2014); Schutz et al. (2018); Yang and Karbstein (2022)
	Rrb1 (57.26 kDa)	Inviable	GRWD1 (49.42 kDa)	48.6% (33.3%)	Rpl3 (43.76 kDa)	80.4% (65.5%)	N-terminal	Iouk et al. (2001)
	Acl4 (42.94 kDa)	Growth defect	-		Rpl4 (39.09 kDa)	63.8% (49.7%)	Internal loop	Pillet et al. (2015)
	Syo1 (70.16 kDa)	Growth defect	HEATR3 (74.58 kDa)	43.6% (26.1%)	Rpl5 (33.72 kDa)	68.7% (50.5%)	N-terminal	Kressler et al. (2012)
					Rpl11 (19.72 kDa)	80.6% (68.3%)	N-terminal	
	Bcp1 (32.66 kDa)	Inviable	BCCIP (35.98 kDa)	47.4% (29.4%)	Rpl23 (14.47 kDa)	87.9% (78.0%)	N-terminal	Ting et al. (2017)
	Sqt1 (47.17 kDa)	Inviable	AAMP (46.75 kDa)	46.1% (28.1%)	Rpl10 (25.36 kDa)	74.6% (63.8%)	N-terminal	West et al. (2005); Yang et al. (2023)
	Nap1 (47.89 kDa)	Growth defect	NAP1L1 (45.37 kDa)	58.4% (36.0%)	Rps6 (27.00 kDa)	75.6% (61.4%)	C-terminal + loop domain	Rossler et al. (2019)
					Rpl39 (6.34 kDa)	86.5% (63.5%)	?	
					Rpl42 (12.21 kDa)	87.2% (74.3%)	?	
	Puf6 (75.11 kDa)	Growth Increased	PUM3 (73.58 kDa)	47.8% (29.7%)	Rpl43 (10.09 kDa)	79.8% (67.0%)	?	Liang et al. (2019)
	Loc1 (23.59 kDa)	Growth defect	-		Rpl43 (10.09 kDa)	79.8% (67.0%)	?	
**Ribosome turnover**	Atg8 (13.63 kDa)	Growth defect	LC3B (14.69 kDa)	58.9% (38.0%)	Rpl12 (17.82 kDa)		N-terminal	Chen et al. (2025)
	Atg11 (135.03 kDa)	Growth defect	-		Rpl12 (17.82 kDa)		Middle domain	
**Ribosome hibernation**	Lso2 (10.50 kDa)	Growth defect	CCDC124 (25.84 kDa)	23.8% (17.9%)	-		-	Wang et al. (2018); Wells et al. (2020)
	Stm1 (30.00 kDa)	Growth defect	SERBP1 (44.97 kDa)	34.1% (22.9%)	Rps3 (26.50 kDa)		Middle domain	Shetty et al. (2023); Van Dyke et al. (2006)
					Rps10 (12.74 kDa)		C-terminal	
					Rps12 (15.47 kDa)		Middle domain	
					Rps18 (17.04 kDa)		C-terminal	
					Rpl11 (19.72 kDa)		N-terminal	

**Table 2.** Homologs of ribosome-associated proteins in *C. albicans*, *C. neoformans*, and *A. fumigatus*

	Target (*S. cerevisiae*)	Homolog (*C. albicans*)	Similarity against human (Identity)	Homolog (*C. neoformans*)	Similarity against human (Identity)	Homolog (*A. fumigatus*)	Similarity against human (Identity)
Ribosome biogenesis	Yar1	O	-	O	-	O	-
	Tsr4	O	41.49% (24.89%)	O	39.50% (26.20%)	O	41.87% (26.25%)
	Tsr2	O	45.26% (28.02%)	O	48.52% (30.38%)	O	48.25% (35.09%)
	Rrb1	O	46.85% (34.41%)	O	58.05% (42.34%)	O	51.78% (39.63%)
	Acl4	O	-	O	-	O	-
	Syo1	O	32.56% (19.35%)	O	47.20% (29.44%)	O	36.22% (21.95%)
	Bcp1	O	46.60% (29.32%)	O	42.98% (26.45%)	O	46.09% (27.25%)
	Sqt1	O	48.07% (31.77%)	O	46.99% (29.71%)	O	43.77% (29.24%)
	Nap1	O	55.56% (36.11%)	O	54.87% (37.17%)	O	58.41% (37.07%)
	Puf6	O	48.51% (29.62%)	O	49.13% (28.65%)	O	51.18% (30.84%)
	Loc1	O	-	O	-	O	-
Ribosome turnover	Atg8	O	55.86% (35.86%)	O	58.91% (37.98%)	O	62.32% (38.41%)
	Atg11	O	-	O	-	O	-
Ribosome hibernation	Lso2	O	51.92% (35.38%)	O	47.64% (34.18%)	-	-
	Stm1	O	34.92% (22.45%)	O	42.21% (30.30%)	O	40.13% (29.93%)
